# One Health Approach to Leptospirosis: Human–Dog Seroprevalence Associated to Socioeconomic and Environmental Risk Factors in Brazil over a 20-Year Period (2001–2020)

**DOI:** 10.3390/tropicalmed8070356

**Published:** 2023-07-07

**Authors:** Natacha Sohn-Hausner, Louise Bach Kmetiuk, Alexander Welker Biondo

**Affiliations:** Department of Cell and Molecular Biology, Federal University of Paraná, Curitiba 80035-050, PR, Brazil

**Keywords:** public health, one health, risk factors, sentinels, zoonosis

## Abstract

Despite being considered a neglected, re-emerging and the most widespread zoonotic disease worldwide, human-dog leptospirosis has not been subjected to One Health approach, and neither were its socioeconomic and environmental risk factors, as well as concomitant spatial analysis over time. Accordingly, notified human leptospirosis cases, incidence rate and urban hotspot areas, in addition to a systematic review of dog leptospirosis cases, were performed nationwide from 2001 to 2020 in Brazil. Data on Gross Domestic Product (GDP), flooding and study areas were also assessed and tabulated. Human–dog leptospirosis cases were simultaneously mapped with overlapping flooding areas, along with the main circulant serovars. Comparative outcome has shown that dogs may be exposed similarly to humans, becoming important sentinels and/or reservoirs for human leptospirosis in larger geographic areas. Moreover, the study herein can help in the decision and implementation of public policies in Brazil and may serve as a model for other tropical countries worldwide.

## 1. Introduction

Leptospirosis, a bacterial infection caused by the genus *Leptospira*, has been considered a neglected and re-emerging disease of global importance for public health, impacting human and animal morbidity and mortality [1]. Considered also the most widespread zoonotic disease worldwide [2], leptospirosis has been characterized by non-pathognomonic signs including abrupt febrile onset, headache, jaundice, vomiting, abdominal pain, and death [3]. Genus *Leptospira* currently comprises ten known pathogenic, five potentially pathogenic and seven saprophytic species [4], divided into more than 300 identified serovars based on the heterogeneity of their structural lipopolysaccharide components [5].

The World Health Organization (WHO) has demanded an increase in leptospirosis surveillance to determine global losses, improve surveillance methods and establish effective measures for disease control and prevention [6]. In addition, the WHO has called for studies focused on the One Health Initiative, combining human, animal, and environmental health in a holistic approach to zoonotic diseases [6]. In such a scenario, a One Health approach may be required for leptospirosis for comprehensive understanding of pathogen occurrence and dynamics and associated social and economic risk factors of affected populations, as recently reported in Brazilian seashore and oceanic islands [7].

Leptospirosis is an important human health concern. Despite still being considered an underreported disease, a systematic review and modeling study has estimated 1.03 million worldwide cases, with around 58,900 deaths every year [8]. The highest global estimated leptospirosis morbidity included South and Southeast Asia, Oceania, the Caribbean, regions of sub-Saharan Africa and Latin America, recently with sporadic cases and alarming outbreaks in Australia [9]. The annual estimated leptospirosis morbidity rate in southern Latin America and Andean ranges from 1.43 to 39.8 per 100,000 habitants, with around 40.2% of the overall reported cases in Brazil alone, which accounts for almost half of the total Latin American population [10]. Notification of leptospirosis cases and disease surveillance are made mandatory by the Brazilian Ministry of Health, with case reports mostly in southern and northern regions [11], annual average of 3734 isolated suspected outbreaks, incidence of 2.1 per 100 thousand inhabitants, and lethality rate of around 8.7% [12].

Environmental considerations are also important with respect to Leptospira transmission and persistence. Despite a commonly incidental leptospirosis host, dogs may also present with a serious disease due to pathogenicity, extended shedding period [13], potential infection during contact with rats, and transmission due their close contact with owners [14]. Not surprisingly, early detection of leptospirosis in dogs may impact human disease control and prevention [14,15]. In addition, transmission may be aggravated by unvaccinated dogs in contact with infected rats, as is common in low-income neighborhoods of endemic countries [16], which may switch the role of dogs from sentinels to reservoirs in the *Leptospira* cycle [17,18,19]. Initially playing a role in the wildlife cycle, *R. norvegicus* and *R. rattus* have spread worldwide and become ubiquitous in anthropized settings, recognized as the most important and abundant source of *Leptospira* pathogenic serovars [20]. In these areas, human-to-human transmission is rarely reported [21], and dogs have been indicated as accidental hosts for most serovars except Canicola, corroborated by an increase in human leptospirosis incidence [22]. Even considering wildlife cycles, native carnivores and rodents are the most frequent hosts for *Leptospira* spp. in Latin America according to a systematic review [23]. Nonetheless, the current role of wildlife as a *Leptospira* spp. source and its impact on urban and rural settings remain to be fully established [24].

Leptospirosis has been an important environmental health concern. *Leptospira* has the ability to live in a wide range of environments and reportedly endure for a very long time [25]. Although *Leptospira* spp. may be transmitted by contact of mucous membranes or skin with urine, blood, and tissues of infected animals, indirect transmission through contaminated soil and water is more likely to occur, as *Leptospira* spp. may survive in soil and water for weeks to months. Moreover, transmission is more favorable in tropical regions [1], mostly in flooding areas by exposure to contaminated water [26], with the urine of infected rats [27], wild [28], or domestic animals [18].

Overall, concomitant serology and spatial analyses performed in human and dog samples may provide a better understanding of associated risk factors, cross-infection, and common environmental exposure of households. Accordingly, the study herein has assessed the notified human leptospirosis cases and their spatial location, the incidence rate in Brazil from 2001 to 2020, and annual correlation with the municipal Gross Domestic Product (GDP). Meanwhile, a systematic review of dog leptospirosis cases was performed, and data on flooding areas gathered and analyzed in the same period. Finally, human–dog leptospirosis cases were mapped together along correspondent environmental factors such as areas of flooding and main circulant dog serovars.

## 2. Materials and Methods

### 2.1. Study Area

The study herein was conducted in all 5570 Brazilian municipalities distributed in the territorial area of 8,510,345,540 km^2^, with an estimated population of 211,755,692 inhabitants in 2020, divided into northern, northeastern, southeastern, southern, and central–western regions [29]. Brazil’s climate is diversified due to its geographic location, territorial extension, and air mass dynamics, directly causing regional and seasonal differences in both temperatures and rainfall. As annual precipitation commonly varies from 387 to 4003 mm, autumn–winter has been characterized as dry and spring–summer as wet periods [30]. The current Brazilian total and per capita GDP has been ranked worldwide as 8th and 82nd, respectively, with 85.5% of households possessing a general water supply network, 68.3% with sanitary sewage and 84.4% with garbage collection [31].

### 2.2. Data Acquisition

The study was carried out considering the Brazilian territory and assessing the historical information and updated data on confirmed cases of human leptospirosis, GDP for each correspondent city, seroprevalence of *Leptospira* spp. in dogs, and flooding areas from 2001 to 2020.

#### 2.2.1. Human Leptospirosis Data

Information on confirmed cases of human leptospirosis from 2001 to 2020 in Brazil was obtained from the Information System on Notifiable Diseases (SINAN) and used to construct the spatial analysis of historical annual incidence [32]. Data were uploaded to this nationwide system by physicians and other healthcare professionals in their own municipalities, including cases and related conditions of leptospirosis and several other mandatory notifiable diseases [32]. A software system (Sinan Net 5.0) made these data available through the Information Technology Department of the Unified Health System [33].

Finally, general population data for each municipality were obtained at the Brazilian Institute of Geography and Statistics (IBGE) and included estimated yearly population and GDP, both used for socioeconomic analysis [34].

#### 2.2.2. Dog Leptospirosis Data

Data on canine leptospirosis in Brazil were obtained by a systematic literature review in English, Spanish and Portuguese in selected electronic databases including Scielo, PMC, Embase, Mendeley, BVS and Scopus using the following search keys: (leptospir*) AND (dog) AND (prevalence OR seroprevalence OR serol* OR seroepidem*) AND (Brazil).

Initial inclusion criteria comprised seroprevalence studies in dogs from Brazil, published in peer-reviewed journals and written in English, Spanish or Portuguese. In a second filtering stage, all duplicated reports and non-peer reviewed studies (i.e., theses, opinion articles, letters to the editor) were excluded. As a final step, in-text references from the selected articles were also screened and retrieved from the literature articles that fulfilled the inclusion criteria from the abovementioned databases or from Google Scholar. All included articles must have reported the number of tested and positive dogs for the overall sampled population and for each condition or exposure factor.

For each article, data on authors, year of publication, city and state, region of the country, sampling year/s, type of dog (domestic, stray, sheltered, other), health status (apparently healthy, with clinical suspicion of leptospirosis), the total number of samples, seropositivity (%) and serovars found were recorded. Relevant information for each article was gathered in a software spreadsheet (Microsoft 365). Article results including sampling years, dog types, health statuses, and city location were considered as an independent study. This means that the same article may have included one or more studies. As vaccination status is highly variable among articles and given the differences among vaccine types, brands, and periods since the last vaccination, it was not considered for analysis herein.

### 2.3. Data Analysis

#### 2.3.1. Spatial Analysis of Human Leptospirosis Cases

For statistical analysis, the incidence of human infection was initially calculated by municipality for each year from 2001 to 2020, with the number of municipality inhabitants recorded as denominator for each reference year. The Local Indicators of Spatial Association (LISA), a statistical parameter that allows the description of the degree of similarity or difference of each event in relation to the next, was calculated for testing the local autocorrelation. Thus, the total LISA sum of all areas was proportional to the value obtained for the index by Moran Global [35]. The LISA index generated a choropleth map (LISA Map) that expressed the spatial dependence at the local level in which the areas were classified as insignificant; low–low: significance of 0.05 (95% confidence); low–high: significance of 0.01 (99% of confidence); high–low: significance of 0.001 (99.9% confidence); high–high: significance of 0.0001 (99.99% confidence) [36]. All analyzes were performed in the R 4.0.4 environment [37].

The GDP was used herein as one of the most used indicators in macroeconomics for quantifying the municipality economic activity during the studied period. A high GDP value may indicate the development of the local economy, low or close to zero indicating no growth in the period and a negative value indicating an ongoing recession process [31]. GDP was also chosen for comparative socioeconomic analysis of municipalities due to its annual assessment, as other indexes such as Human Development Index (HDI) and Social Vulnerability Index (SVI) have been generated from demographic censuses every 10 years, last available in 2010 [38]. The correlation between the Incidence Rate and the GDP of each municipality per year was calculated in an environment R 4.0.4 [39] with a significance level of 5%.

#### 2.3.2. Spatial Analysis of Canine Leptospirosis

Canine serosurvey studies were classified by prevalence as high (>30%), medium (>15% and <30%), and low (<15%) and plotted according to sampling city; the most common serovars found were also spatialized in the meta-analysis. The results were plotted on a comprehensive map using the R Program [39].

#### 2.3.3. Spatial Analysis of Human Leptospirosis, Canine Leptospirosis, and Environment

The vulnerability of flooding areas obtained from the National Water and Sanitation Agency [40] were superimposed with the history of human leptospirosis cases (2001–2020) and with the history of seropositivity of dogs (2001–2020) and/or the most frequently found serovars in dogs for comparison. Thematic maps were created in the R 4.0.4 environment [39].

## 3. Results

### 3.1. Human Leptospirosis Cases from 2001 to 2020

From 1 January 2001 to 31 December 2020, a total of 71,141 human leptospirosis cases were registered in the SINAN system, Brazil. The total distribution of confirmed cases by municipality residence was gathered and presented (Figure S1). Overall, large urban centers presented the highest prevalence during the period, including mostly state capitals along with other major cities in the northern, northeastern, central–western, southeastern, and southern regions.

### 3.2. Dog Leptospirosis Studies and Cases from 2001 to 2020

The bibliographical survey initially resulted in a total of 686 scientific articles, 71 of which were included in the present review following the screening based on inclusion criteria (Figure S2). Of the selected articles, 138 records (information) were found in 81 municipalities of 19 different Brazilian states. The cities with the highest number of records were São Paulo—SP (11 records) Londrina—PR (10 records), Curitiba—PR (9 records), and Botucatu—SP (7 records). These cities were located in the southeastern and southern Brazilian regions. Other cities had up to four records and the majority (59 municipalities) presented only one record (Tables S1 and S2).

### 3.3. Correlation between Incidence Rate and GDP in Brazil from 2001 to 2020

**Figure 1:**
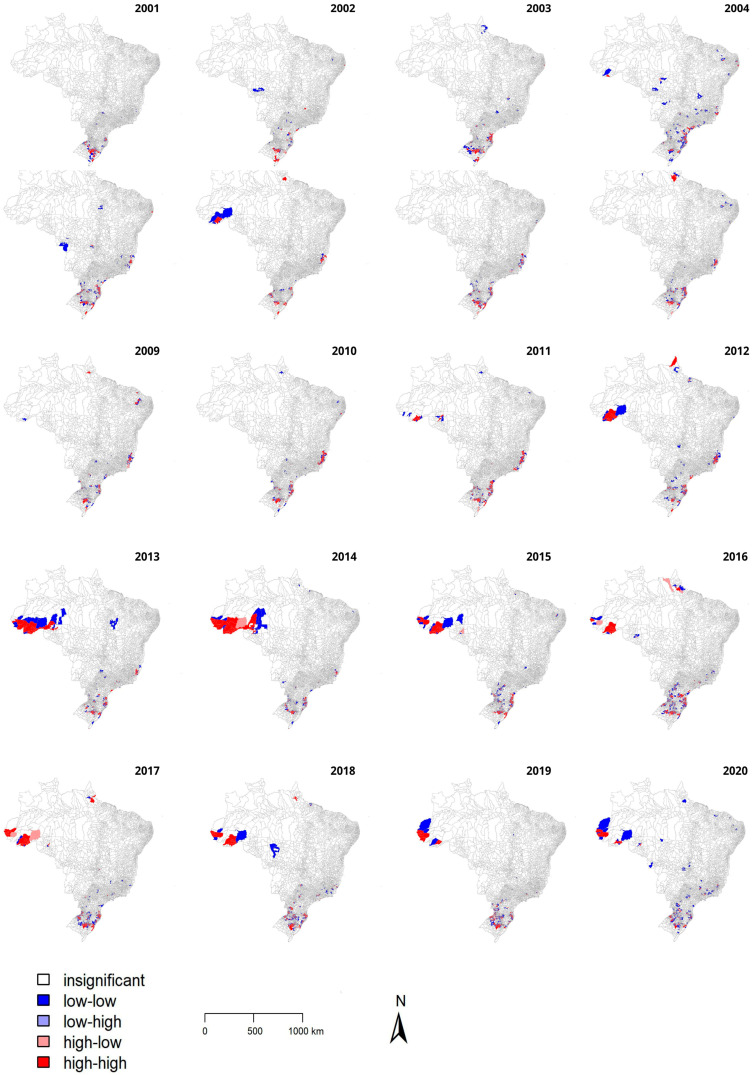
Results of Local Indicators of Spatial Association (LISA) for human leptospirosis in Brazil from 2001 to 2020. Clusters and spatial outliers (confidence interval between 95 and 99.9%), as follows: Insignificant (white): cities were not statistically significant; low–low (blue): significance of 0.05 (95% confidence)—city with low incidence of leptospirosis and low average considering neighboring cities; low–high (light blue): significance of 0.01 (99% of confidence)—city with low incidence of human leptospirosis and high average considering neighboring cities; high–low (pink): significance of 0.001 (99.9% confidence)—city with high incidence of human leptospirosis and low average considering neighboring cities; high–high (red): significance of 0.0001 (99.99% confidence)—city with high incidence of human leptospirosis and high average considering neighboring cities.

The incidence rate of human leptospirosis per 100,000 inhabitants ranged from around 1.5 to 2.5 during the period, with the highest incidence rates in 2001, 2006, 2011 and 2014 (Figures S1 and S3 and Table S3). The incidence rate has shown a high local spatial association in the southern, southeastern, and northern region municipalities during the period, with results shown by the Local Indicators of Spatial Association (LISA) map (Figure 1).

In general, municipalities with a high incidence rate of human leptospirosis in the southern and southeastern regions showed high spatial correlation (red areas) throughout the entire period, while municipalities in the northern region presented high spatial correlation as of 2011. The central region of Brazil, on the other hand, showed high significance for non-spatially clustered cases (blue areas).

**Figure 2:**
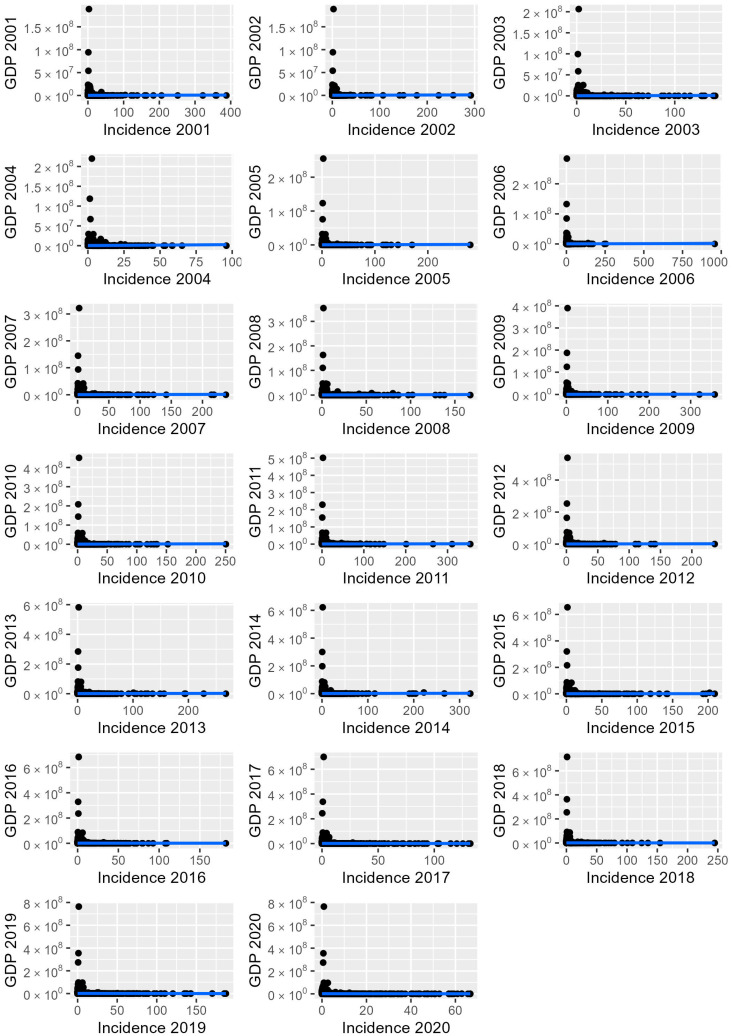
Correlation between *Leptospira* spp. incidence rate in humans and GDP in Brazil from 2001 to 2020. Blue line indicate linear tendency and black circles represents the data set of GPD and incidence of human leptospirosis for each 5,570 Brazilian municipalities.

The correlation between the leptospirosis incidence rate and the GDP was assessed and results showed a significant but weak positive correlation (0.28 to 0.37) for all years (Figure 2).

### 3.4. Spatial Distribution of Canine Seroprevalence and Main Serovars

**Figure 3:**
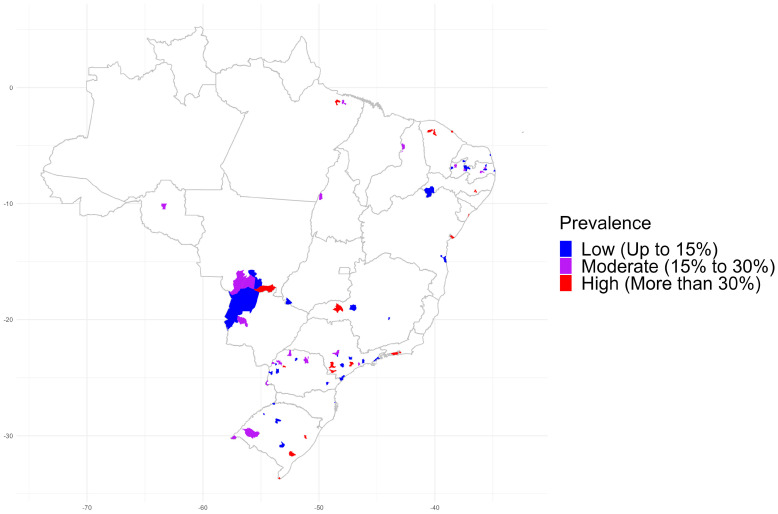
Seroprevalence of *Leptospira* spp. in dogs of Brazil, systematic review from 2001 to 2020.

Seroprevalence in dogs was divided and presented in a thematic map as low (<15%), medium (between 15% and 30%) and high (>30%) values (Figure 3).

Results showed a high number of *Leptospira* spp. serosurvey studies in southern, southeastern, and northeastern cities, and a few in northern Brazil, with a high dog prevalence in state capitals such as Porto Alegre, São Paulo, Rio de Janeiro, Salvador, Aracaju, Fortaleza and Belém.

**Figure 4:**
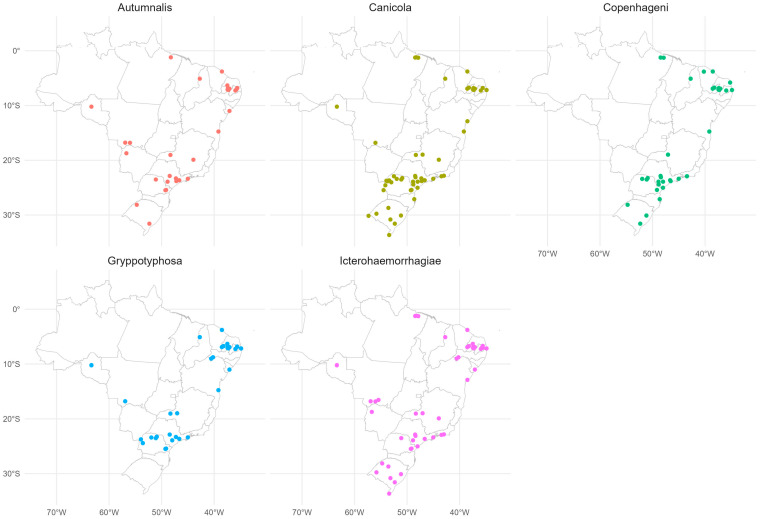
Most frequent *Leptospira* serovars in dogs in Brazil from 2001 to 2020, and the respective study sites. Red dots are seropositive dogs for Atumnalis serovar, olive green dots for Canicola serovar, emerald green dots for Copenhageni, blue dots for Grippotyphosa and violet dots Icterohaemorrhagiae.

The meta-analysis review showed that from 2001 to 2020 in Brazil, the most prevalent serovars in dogs were Autumnalis, Canicola, Copenhageni, Grippotyphosa and Icterohaemorrhagiae, occurring in several Brazilian states (Figure 4).

### 3.5. Spatial Clusters

**Figure 5:**
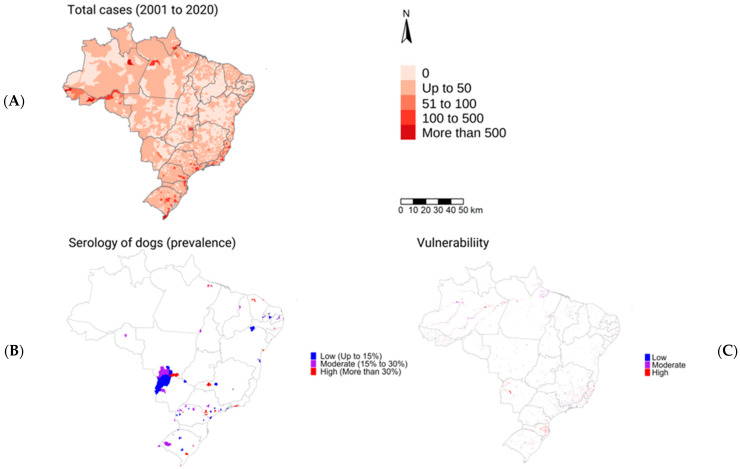
Incidence of *Leptospira* spp. in humans of Brazil from 2001 to 2020 (**A**); seroprevalence of anti-*Leptospira* spp. antibodies in dogs in areas with flooding risk from 2001 to 2020 (**B**); areas of vulnerability to flooding in Brazil (**C**).

A single map was constructed to overlap the incidence of human leptospirosis, the anti-*Leptospira* spp. antibodies in dogs and the areas of vulnerability to flooding in Brazil (Figure 5). In this map, regions with the highest dog seropositivity were associated to those with the highest human incidence and within or nearby areas of flooding risk.

## 4. Discussion

To the authors’ knowledge, this was the first concomitant and comprehensive approach analyzing a 20-year period of human, dog, socioeconomic and environmental risks for leptospirosis in Brazil. Although the study herein provided a holistic understanding of leptospirosis, associated risk factors for human beings have long been established. Brazilian urban areas, as most developing countries worldwide, have been historically associated to disorganized settings of major cities, with inadequate sanitary conditions and poverty, living in flooding areas with rats and consequent leptospirosis outbreaks [25,41,42,43,44].

The present study demonstrated that leptospirosis has been endemic throughout the Brazilian territory, with predominance in the southern, southeastern, and northern regions, mainly in major urban areas, with a high incidence of human cases in the last 20 years (Figure S1), probably due to better diagnosis, surveillance, and reporting [41,45,46,47]. Such risk for human infection in the southern Brazilian region has already been described in relation to environmental, socioeconomic, and animal (livestock) variables in the Rio Grande do Sul state [41]. Despite an eightfold higher human incidence in rural than in urban populations, infection was associated to river ecoregion, soil contents and tobacco and rice crops, with urban cases concentrated in the state capital [41].

Human leptospirosis in Brazil has shown a positive autocorrelation in these regions by the Lisa map, indicating a spatial association and tendency of nearby cities. Expectedly, human leptospirosis presented positive autocorrelation indicating a spatial association at the Lisa Map in such regions, with a similarity between the attribute values of geographically neighboring cities. In addition, the Lisa Map provided a spatial view of leptospirosis cases, identification of clusters, and evaluation of the significance level of spatial association. Based on such approach, the southern Brazilian region was the most important cluster in the early 2000s, followed by the southeastern region until 2011, and then, later, by the northern, central–western, and northeastern Brazil. The Lisa Map offered spatial data analysis, extracting additional information, not directly noticeable by usual visualization and classification techniques. A recent study applying the Lisa Map in Thailand successfully showed hotspots and clusters in rice crops as important areas for human leptospirosis, which were areas of greater importance for intervention and public health prevention [27].

Although a low correlation was observed between GDP and leptospirosis incidence (<39), the overall socioeconomic status and income may not reflect the existing intra-municipal inequalities, particularly observed in developing countries such as Brazil. GDP measures the overall production flow per year but may fail to include the differences in income distribution among poor and rich neighborhoods within city, quality of life (including environmental quality) and consumption [31]. Despite the fact that Human Development Index (HDI) and Environmental Performance Index (IDA) may be used for decision-making at the city, state, and country levels, such indexes may be insufficient for health analysis. Recently, a One Health Index has been proposed as a holistic index for future comparisons, considering human, animal, and environmental health in its calculation [31]. Due to the complex environmental transmission pathways of leptospirosis, as previously established [48], the use of holistic indexes for comparison may be useful for detecting vulnerability areas to leptospirosis.

Another epidemiological strategy for understanding leptospirosis is the detection of most prevalent serovars, as well as the hosts that participate in the disease cycle [49]. *Leptospira* spp. may persist in natural niches, circulating in primary hosts, wild rodents, from which they reach other populations of synanthropic and or domestic animals as secondary hosts. In such a scenario, high numbers of domestic animals associated to ecosystem modifications may result in new pathways and spreading of *Leptospira* spp. in the environment [50]. In urban areas, dogs have been indicated as a potential source in leptospirosis transmission due to their close relationship with human owners [51].

Based on the systematic review of the last 20 years on the seroprevalence of *Leptospira* ssp. in dogs in Brazil, prevalence data were updated [51,52] and the main circulating serovars nationwide were mapped. Although direct *Leptospira* transmission from dogs to humans has been reportedly uncommon [14,53], high environmental exposure to pathogenic *Leptospira* may suggest dogs as disease sentinels and an epidemiological link between human and environmental sources [54,55,56]. Thus, concomitant human and dog serosurvey, along with individual case characteristics and spatial analysis, may be a better assessment of risk factors and common environmental exposure, providing basis for effective prevention strategies and control measures, particularly in areas considered of greater risk and reduced financial resources.

Although published studies on leptospiral seroprevalence in dogs herein covered 19/27 (70.4%) Brazilian states and only 81/5570 (1.45%) municipalities, the results of state capitals and other major cities allowed to spatially identify higher prevalence in dogs living in urban areas, such as São Paulo, Rio de Janeiro, Salvador, Fortaleza, Belém, Porto Alegre, and Aracaju (>30%), the 1st, 2nd, 4th, 5th, 11th, 12th, and 33rd largest Brazilian cities, respectively. Most human leptospirosis cases were also located in state capitals and major urban cities, such as São Paulo, Rio de Janeiro and Salvador, where large leptospirosis outbreaks were recorded after flooding, corroborating that exposure of dogs has been positively related to human infection, probably and mainly in places with high human (and dog) density, associated to low income, nearby rivers, infested by rodents, lacking housing and infrastructure [57,58,59]. Not surprisingly, dog exposure to disease has been positively related to human infection, as dogs may be considered environmental sentinels for leptospirosis [60,61], including on a local scale [62].

Regarding dog serovars, icterohaemorrhagiae and copenhageni have been frequently associated to severe human cases in Brazil [11,57], corresponding with the role of dogs as reservoirs of main serovars in urban areas and the importance of canine surveillance and monitoring. In Africa, a systematic review found three major predominant species (*Leptospira borgpetersenii*, *L. interrogans* and *L. kirschneri*) in human and animal infections, indicating that cattle may be important hosts, but few data were available at the time to allow comparison linking human and animal populations [18]. Though this study did not include environmental factors, the authors advocate a One Health approach to improve understanding of animal-to-human leptospirosis transmission on the African continent. Similar conclusions were presented for animal infections in Ecuador, distributed in mainland and insular areas, posing a significant human health risk [63].

A series of studies worldwide have shown that exposure to water, with few exceptions, was associated to a higher risk of human leptospirosis [48] and increasing infection likelihood in dogs by 68% [64]. The highest incidence of human leptospirosis herein was observed in the years 2001, 2006, 2011 and 2014, characterized by a 5-year interval, similar to the cyclic pattern of the El Niño–Southern Oscillation (ENSO), the most relevant phenomenon for the interannual climatic variability on a global scale [65,66]. The ENSO has directly influenced temperature and precipitation in South America, increasing and decreasing river flow, flooding, drought at the regionalized level [67]. In Brazil, the ENSO impact of the hot (El Niño) and cold (La Niña) phase has mainly influenced the rain precipitation in southern, northern, and northeastern Brazilian regions. In El Niño years, rain precipitation exceeded the average climatologic levels in the southern region while staying below average in northern and northeastern regions; rain precipitation inversely occurred in these regions in La Niña years [67].

The flooding map has also superposed human and dog leptospirosis; as such, triple overlapping altogether may be intimately connected. Recent occurrence of precipitation extremes has increased and may continue to increase as a result of climate change and has been considered a concern in urban areas, including with environmental, socioeconomic and health consequences, particularly of water-borne pathogens such *Leptospira* spp. [68]. In such a scenario, flooding mitigation in urban areas may require structural changes such as increase in drainage and vegetation cover, warning systems [69], and pathogen detection and surveillance in humans and dogs, which altogether effectively prevent leptospirosis in a One Health perspective.

Animal leptospirosis in Latin America and the Caribbean countries has been linked through multiple factors in the animal–human–ecosystem interface, showing a wide distribution of outbreaks and tropical terrestrial biomes as the predominant ecosystems [70]. As observed herein, despite being limited to a few countries, occurrence of leptospirosis cases and epidemic outbreaks is related to climatic and ecological factors, with 6 of 11 known *Leptospira* pathogenic species found in the region. Although this study concludes that leptospirosis prevention and control measures should consider animal and human health issues in the ecosystem’s context, the territorial country land borders and trade, no human and environmental reviews are provided.

Companion animal leptospirosis has long been considered an archetypal One Health issue, requiring a thorough analysis of pathogenic leptospiral strains, host species, and environment, including vaccine design, prophylaxis efforts and educational programs, providing basis for policy development [71]. As observed herein, regional distribution of different leptospiral strains provided specific immune protection of companion animals, particularly by advances on vaccine technology [71]. Moreover, molecular detection identified reservoir host species and environmental sources; however, besides characterization of clinical onsets and outbreaks, no concomitant human–dog–environment approach has been indicated [71].

In regard to limitations in the present study, the data on human leptospirosis cases were obtained at the Brazilian Ministry of Health, based on mandatory notification, and relying on the inclusion of cases in the surveillance system by local health professionals nationwide [72]. As a result, cases may have been underreported and possibly under- or misdiagnosed, particularly in regions with inadequate financial and human resources for diagnosis and proper report through the notification system [73,74]. In addition, as previously reported, patients with mild or non-specific symptoms may refuse to seek health care or be wrongly diagnosed with other endemic tropical febrile syndromes such as dengue and malaria [75,76]. Also, confirmative diagnosis for human and animal leptospirosis has been based mostly on the Microagglutination Test (MAT), which may fail to detect early infection, low titers, and unusual strains, particularly in areas with low risk or without previous information [77]. As another limitation, dog leptospirosis herein was limited to the relatively few published serosurveys and municipalities during the period. The lack of current information may highlight the importance of further survey studies in known and unknown areas to establish the leptospirosis prevalence and dynamics over time, if possible, with owners and their dogs in the same households. Finally, lack of wildlife (i.e., rodents) and livestock data herein should be considered as a limitation, and further studies should be conducted to fully establish the role of other mammal hosts in the leptospirosis cycle and dynamics over time.

The findings herein highlighted the importance of the issue and demanded a One Health governance approach with a specific National Strategic Plan to leptospirosis, as also proposed in Ecuador [64], the Caribbean [71], Latin America [42], India [78], Thailand [79], Fiji [80], Australia [9], and Africa [63]. One Health approach has been critically useful and essential to address all connected issues in leptospirosis including diagnosis, treatment, control, monitoring, and prevention [9]. Finally, effectiveness and ethical principles should be conducted altogether and reflect the real economic, social, and political settings, as One Health approach has been implemented for control of leptospirosis and several other infectious diseases in such scenarios [9].

## 5. Conclusions

Leptospirosis is an important global public health problem occurring at the animal–human–environment intrinsic interface. Despite likely underdiagnosis, results herein showed such concomitant occurrence, highlighting the importance of a One Health approach on leptospirosis, as human and dog case distribution herein overlapped nationwide flooding areas in a recent 20-year period. Although with few available studies, dogs may be used as environmental sentinels for human leptospirosis. Finally, official national databases in Brazil and other endemic countries worldwide should gather, map and present both human and dog seroprevalence in real time, along with rainfall distribution, as a One Health approach for easy visualization of hotspots, dynamics and serovar distribution, at different geographic levels including cities, metropolitan areas, states, and regions.

## Data Availability

Not applicable.

## Supplementary Materials

The following supporting information can be downloaded at: https://www.mdpi.com/article/10.3390/tropicalmed8070356/s1, Figure S1: Incidence rate of human leptospirosis in Brazil from 2001 to 2020; Figure S2: Flow used to search for articles on the seroprevalence of Brazilian dogs; Table S1: Result of the seroprevalence review of Brazilian dogs; Table S2: Articles of the seroprevalence review of Brazilian dogs; Figure S3: Incidence rate of human leptospirosis per 100,000 inhabitants in Brazil from 2001 to 2020 and Table S3: Correlation between Incidence Rate and GDP, Brazil (2001 to 2020).
