# Supplementary material for: One Health Approach to Leptospirosis: Human–Dog Seroprevalence Associated to Socioeconomic and Environmental Risk Factors in Brazil over a 20-Year Period (2001–2020)
Source: Trop Med Infect Dis. 2023 Jul 7;8(7):356. doi: 10.3390/tropicalmed8070356 (PMC10383893; doi:10.3390/tropicalmed8070356)
Supplement: Supplementary file 1 [file tropicalmed-08-00356-s001.zip › tropicalmed-2435462-supplementary.pdf]

Table S1. Result of the seroprevalence review of Brazilian dogs

Database	Results	Duplicated	Non relevant	Relevant	old data	Missing data	Unavailable	used
Scielo	5	1	0	4	0	0	0	4
Pub Med Central (PMC)/Medline	70	0	35	35	7	2	2	24
Embase (Elsevier)	61	0	29	32	6	0	0	26
Mendeley	257	14	173	70	10	0	1	59
BVS (Lilacs)	42	0	3	32	3	0	0	29
Scopus (Elsevier)	103	0	38	65	10	2	0	53
Total Database	*538*	*15*	*278*	*238*	*36*	*4*	*3*	*57 different studies*
Google Academic	148	0	0	0	15	0	0	133
Total Google	*148*	*0*	*0*	*0*	*15*	*0*	*0*	*14 different studies*
**Final number of articles that entered the meta-analysis**								**71 articles**
					Total number of records found in articles			*138 records*
					Total number of Brazilian cities registered			*80 cities*

Table S2. Articles of the seroprevalence review of Brazilian dogs

REFERENCE	CITY	NUMBER OF SAMPLES	POSITIVITY (%)	YEAR OF SAMPLE COLLECTION	TYPE OF DOG	SEROVAR
Abreu et al., 2019	Barra do Quaraí - RS	32	21,88	2013-2014	domiciled	Australis, Butembo, Canicola, Cynopteri and Panama
	Cerro Largo - RS	127	8,66			Australis, Autumnalis, Bratislava, Butembo, Cynopteri, Copenhageni, Icterohaemorrhagiae, Hardjo and Panama
	Derrubadas - RS	33	3,03			Pomona
Aguiar et al., 2007	Monte Negro - RO	173	30,6	uninformed	domiciled	Autumnalis, Pyrogenes, Canicola, Shermani, Butembo, Hardjo, Bratislava, Grippotyphosa and Icterohaemorrhagiae
		156	23,7			Autumnalis, Pyrogenes, Canicola, Shermani, Butembo and Icterohaemorrhagiae
Albuquerque et al., 2020	Belém - PA	145	64,14	2018	shelter	Djasiman, Canicola, Cynopteri, Icterohaemorrhagiae, Seramanga, Sejroe, Pyrogenes, Australis, Shermani and Celledoni
Azevedo et al., 2011	Patos - PB	152	19,73	2008	domiciled and treated in clinics/laboratories	Autumnalis, Grippotyphosa, Castellonis, Icterohaemorrhagie, Australis, Hebdomalis and Butembo
Batista et al., 2004	Patos - PB	130	20	2003	wanderer	autumnalis, pomona, grippotyphosa, patoc, australis, cynopteri, javanica, icterohaemorrhagiae, tarassovi, andamana, shermani, butembo and hebdomadis
Batista et al., 2005	Campina Grande - PB	285	21,4	2003	domiciled	Autumnalis, Copenhageni and Canicola
Benitez et al., 2012	Jataizinho - PR	653	20,21	2010	domiciled	Canicola, Butembo, Pyrogenes, Grippotyphosa, Ballum, Bratislava, Copenhageni, Pomona and Tarassovi
Benitez et al., 2021	Londrina - PR	729	21,26	2015-2016	domiciled	Canicola, Butembo, Bratislava, Grippothyphosa, Copenhageni, Icterohaemorrhagiae, Pomona and Pyrogenes
Bernardino et al., 2021	Alagoa Grande - PB	100	7	2017	domiciled	Icterohaemorrhagiae
	Alagoa Nova - PB	65	9,6			Icterohaemorrhagiae, Autumnalis and Pomona
	Areia - PB	79	20,2			Icterohaemorrhagiae, Autumnalis and Serjoe
	Bananeiras - PB	72	6,9			Icterohaemorrhagiae
	Borborema - PB	17	10			Autumnalis, Pyrogenes and Canicola
	Matinhas - PB	14	15			Icterohaemorrhagiae
	Pilões - PB	23	20			Icterohaemorrhagiae, Grippotyphosa and Pomona
	Serraria - PB	21	28			Icterohaemorrhagiae, Autumnalis and Grippotyphosa

Bier et al., 2013	Curitiba - PR	378	27,8	2009-2010	domiciled	unformed
Blazius et al., 2005.	Itapema - SC	590	10,5	2000-2005	wanderer	Pyrogenes, Canicola and Copenhageni
Brasil et al., 2018	João Pessoa - PB	384	11,7	2015-2016	domiciled and treated in clinics/laboratories	Icterohaemorrhagiae, Grippotyphosa, Canicola, Djasiman and Pomona
Brod et al., 2005	Pelotas - RS	105	52,4 (71,4 for tande)	2001	shelter	Illini, Bratislava, Copenhageni, Sejro, Autumnalis, Van Tienem, Bataviae Swart, Ictero. Kantorovic, Ictero. Verdum and tande (replaces serovars Canicola, Ballum, Icterohaemorrhagiae RGA, Bratislava, Copenhageni, Icterohaemorrhagiae Kantorovic and icterohaemorrhagiae Verdum)
Caldart et al., 2015	Londrina - PR	24	45,8	2004	domiciled	Bratislava, Butembo, Hardjo and Pyrogenis
		38	44,7	2005		Autumnalis, Bratislava, Butembo, Canicola, Copenhageni, Hardjo, Icterohaemorrhagiae and Pyrogenis
		1	0	2006		none
		17	17,6	2007		Bratislava, Copenhageni and Pomona
		50	10	2008		Butembo, Copenhageni, Icterohaemorrhagiae and Pomona
		39	10,3	2009		Autumnalis, Bratislava and Icterohaemorrhagiae
		25	20	2010		Butembo, Canicola and Pyrogenis
		30	13,3	2011		Butembo, Canicola and Hebdomadis
		13	16,7	2012		Copenhageni
Castro et al., 2011	Uberlândia - MG	268	28,4	2008	domiciled	Autumnalis, Tarassovi, Canicola, Grippotyphosa, Bratislava, Icterohemorragiae, Australis, Pomona and Wolffi
Castro et al., 2011	Uberlândia - MG	150	38	2008	domiciled	Autumnalis, Bratislava, Canicola, Tarassovi, Pomona, Icterohaemorrhagiae, Grippotyphosa, Hardjo, Wolffi and Australis
Castro et al., 2015	Uberlândia - MG	268	28,4	2008	domiciled	Autumnalis, Tarassovi, Canicola, Grippotyphosa, Bratislava, Icterohemorragiae, Australis, Pomona and Wolffi
Coiro et al., 2011	Botucatu - SP	302	7.6	2011	domiciled	Canicola, Pyrogenes, Hardjoe and Djasiman
Cortez et. al, 2020	Apiaí - SP	41	40,5	2014-2016	domiciled	Bratislava, Castellonis, Canicola, Copenhageni and Pyrogenes
	Cananeia - SP	52	7,1			Canicola, Hebdomadis, Copenhageni, Icterohaemorrhagiae and Cynopteri

	Itapeva - SP	202	42,6			Australis, Bratislava, Autumnalis, Butembo, Castellonis, Canicola, Cynopteri, Hebdomadis, Copenhageni, Icterohaemorrhagiae, Panama, Pomona, Pyrogenes, Wolffi and Andamane
	Itu – SP	277	5,1			Australis, Bratislava, Autumnalis, Canicola, Cynopteri and Grippotyphosa
Cunha et al., 2019	Curitiba - PR	15	20	unformed	domiciled	Gryppotyphosa
Cunha et al., 2022	Curitiba - PR	264	6,1	2017	domiciled	Copenhageni, Pirogenes and Pomona
de Souza Rocha et al., 2022	Santa Bárbara do Pará - PA	51	39,2	2014	domiciled	Canicola, Samaranga, Bataviae, Icterohaemorrhagiae and Djasiman
		68	22,05	2015		Cynopteri, Sejroe, Celledoni, Djasiman, Autumnalis, Ballum, Pyrogenes and Tarassovi
dos Santos et al., 2017	Lagoa Grande - PE	53	7,5	2014-2015	domiciled	Andamana, Australis, Butembo, Grippotyphosa, Icterohaemorrhagiae, Pyrogenes and Patoc
	Petrolina - PE	56	10,7			Andamana, Australis, Butembo, Grippotyphosa, Icterohaemorrhagiae, Pyrogenes and Patoc
	Serra das Confusões National Park - PI	71	0	2013		none
Dreer et al., 2013	Umuarama - PR	175	20	2011	shelter	Canicola, Bratislava, Tarassovi, Hardjo and Pyrogenes
Félix et al., 2020	Pelotas - RS	221	29	unformed	wanderer	Canicola and Icterohaemorrhagia (analysis only for these two)
Fernandes et al., 2013	Natal - RN	365	6,8	2011	domiciled and treated in clinics/laboratories	Shermani, Sentot and Copenhageni
Fernandes et al., 2018	Cajazeiras - PB	125	8,8	2013-2014	domiciled and treated in clinics/laboratories	Icterohaemorrhagiae, Copenhageni, Bratislava, Canicola, Pomona, Grippothyphosa, Australis, Castellonis and Bataviae
	Campina Grande - PB	249	10.8			
	João Pessoa - PB	338	7.1			
	Patos - PB	206	7.7			
	Sousa - PB	125	15,2			
Fernandes et al., 2018	Brejo do Cruz - PB	200	14	2014-2015	domiciled	Icterohaemoragiae, Pomona, Grippotyphosa, Canicola, Autumnalis, Cynopteri, Tarassovi and Wolfii
Fonzar & Langoni, 2012	Maringá - PR	335	12,2	2006-2008	wanderer	Pyrogenes, Canícola, Copennhageni, Bratislava, Gryppotyphosa, Hardjo and Pomona

Freire et al., 2007	Rio de Janeiro - RJ	120	73,3	2004	domiciled and treated in clinics/laboratories	Icterohaemorrhagiae, Copenhageni and Canicola
Furtado et al., 2015	Caiman Ecological Refuge and Barranco Alto Ranch - MS	29	24,1	2008-2010	domiciled	uninformed
	Cantao State Park – TO	56	16,1			
	Emas National Park – GO	83	7,2			
Hafemann et al., 2018	Assis Chateaubriand – PR	35	2,86	2015	shelter	Grippotyphosa
	Marechal Cândido Rondon - PR	49	12,24			Canicola, Butembo and Fortbragg
	Moreira Sales - PR	15	46,67			Canicola and Butembo
	Paranavaí - PR	21	19,05			Canicola and Butembo
	Pérola - PR	23	8,70			Canicola
	São Jorge do Patrocínio - PR	23	26,09			Canicola, Grippotyphosa and Pomona
	Umuarama - PR	15	26,67			Canicola
Jorge et al., 2011	Barão de Melgaço – MT	103	17,48	2002-2006	domiciled	Pyrogenes, Autumnalis, Canicola, Hebdomadis, Sentot, Wolffi, Hardjo and Icterohaemorrhagiae
Jorge et al., 2017	Pelotas - RS	1176	uninformed	2003-2007	domiciled	Canicola, Copenhageni, Ballum and Butembo
Kikuti et al., 2012	Botucatu - SP	1195	20,8	2003-2010	domiciled and treated in clinics/laboratories	Canicola, Copenhageni and Icterohaemorrhagiae
Langoni et al., 2015	Botucatu - SP	151	39,1	2014	shelter	Copenhageni, Djasiman, Icterohaemorrhagiae, Gryppotyphosa, Canicola, Australis, Bratislava, BTU (Botucatu)
Latosinski et al., 2018	Botucatu - SP	106	5.7	2014-2015	domiciled	Canicola, Autumnalis, Grippotyphosa
Lavinskya et al., 2012	Ilhéus - BH	282	7,1	2008	domiciled	Copenhageni, Bratislava, Canicola, Gryppotyphosa, Patoc, Autumnalis, Javanica, Cynopteri and Djasiman
Lemo et al., 2012	Aracaju - SE	100	37	2006-2007	wanderer	Autumnalis, Andamana, Hardjo, Icterohaemorrhagiae, Pyrogenes, Grippotyphosa, Shermani, Tarassovi and Bataviae
Lemos et al., 2020	Patrocínio - MG	241	13,2	2017	domiciled	Copenhageni, Canicola, Icterohaemorrhagiae, Grippotyphosa, Pomona, Tarassovi, Butembo and Hardjo

Magalhães et al., 2006	Belo Horizonte - MG	2589	10,35	2001-2002	domiciled	Canicola, Ballum, Pyrogenes, Icterohaemorrhagiae, Autumnalis, Pomona, Australis and Tarassovi
		828	21,74		wanderer	
Magalhães et al. 2007	Belo Horizonte -MG	3417	13,1	2001-2002	domiciled and wanderer	Canicola, Ballum, Pyrogenes, Icterohaemorrhagiae, Autumnalis, Pomona, Australis and Tarassovi
Mantovan et al., 2021	Pardinho - SP	181	3,87	2019	domiciled	Canicola, Icterohaemorrhagiae, Copenhageni and Pyrogenes
Martins et al., 2013	Fortaleza CE	37	32,4	2011-2013	domiciled and treated in clinics/laboratories	Copenhageni, Icterohaemorrhagiae, Cynopteri, Andamana, Grippotyphosa, Castellonis, Canicola, Serjoe, Wolffi, Autumnalis, Tarassovi and Shermani
Martins et al., 2013	Pinhais - PR	228	14,4	2009	domiciled	Australis, Canicola, Copenhageni, Pyrogenes, Grippotyphosa and Icterohaemorrhagiae
		90	38,9	2010		Canicola, Icterohaemorrhagiae, Pyrogenes, Australis, Autumnalis, Pomona, Bratislava, Copenhageni, Cynopteri and Grippotyphosa
Mascolli et al., 2016	Ibiúna - SP	570	32,8	2007-2008	domiciled	Pyrogenes, Autumnalis and Canicola
Miotto et al., 2018	São Paulo - SP	33	55.5	2013-2016	domiciled and treated in clinics/laboratories	Icterohaemorrhagiae, Australis, Pomona, Butembo, Castellonis, Canicola, Shermani, Cynopteri, Autumnalis, Pyrogenes and Sejroe
Miotto et al., 2018	Mogi das Cruzes - SP	24	0	uninformed	wanderer	none
	São Paulo - SP	7	85,71			Grippotyphosa, Autumnalis, Pomona, Icterohaemorrhagiae, Canicola and Sejroe
		92	51			Autumnalis, Icterohaemorrhagiae, Pomona, Pyrogenes, Canicola, Wolffi and Shermani
Modolo et al., 2006	Botucatu - SP	775	15.3	uninformed	domiciled	Australis, Bratislava, Autumnalis, Canicola, Copenhageni, Icterohaemorrhagiae, Pomona, Pyrogenes and Hardjo
Morikawa et al., 2015	Curitiba - PR	378	9,3	2009	domiciled	uninformed
		286	16,4	2010		Canicola, Icterohaemorraghiae, Gryppotyphosa, Autumnalis, Australis, Pyrogenes, Djasiman and Pomona
		235	10,6			Canicola, Icterohaemorraghiae, Gryppotyphosa, Autumnalis, Australis and Pyrogenes
		189	19			Canicola, Icterohaemorraghiae, Gryppotyphosa, Autumnalis and Australis
		145	13,8			Canicola
Oliveira et al., 2012	Porto Alegre - RS	155	40,6	2007-2009	domiciled	kirschneri, Canicola, Icterohaemorrhagiae and Copenhageni
		65	53,8		wanderer	Canicola, Icterohaemorrhagiae and Copenhageni

		33	72,7		domiciled and treated in clinics/laboratories	Canicola, Icterohaemorrhagiae and Copenhageni
Paz et al, 2015	Belém - PA	130	16,9	2009-2010	domiciled and shelter	Canicola, Patoc, Icterohaemorrhagiae and Copenhageni
	Castanhal - PA	144	17,4			
Paz et al., 2021	Salvador - BH	57	70,18	uninformed	domiciled and treated in clinics/laboratories	Icterohaemorrhagiae, Australis, Canicola, Pomona, Djasiman, Serjoe and Cynopteri
Pinto-Ferreira et al., 2019	Foz do Iguaçu - PR	649	23,11	2014	domiciled	Canicola, Bratislava and Butembo
Rodrigues et al., 2007	São Paulo - SP	20	80	uninformed	domiciled and treated in clinics/laboratories	Copenhageni, Icterohaemorrhagiae, Hardjobovis, Autumnalis, Bratislava, Butembo, Pyrogenes, Hardjoprojitno, Wolffi, Canicola, Grippotyphosa, Patoc, Sentot, Castelonis, Cynopteri and Hebdomadis
Sant'anna et al., 2017	São Gonçalo - RJ	131	32,1	uninformed	domiciled	Icterohaemorrhagiae and Canicola
Santos et al., 2019	Curitiba - PR	15	0	2015-2016	domiciled	none
Santos et al., 2021	São Paulo - SP	31	64,5	2018-2019	domiciled and treated in clinics/laboratories	Autumnalis, Icterohaemorrhagiae, Canicola, Bratislava, Australis, Shermani, Pyrogenes, Pomona, Grippotyphosa, Cynopteri, Bataviae, Ballum, Butembo and Bratislava
Scandura et al., 2020	São Bernardo do Campo - SP	10	28,57	2019	domiciled and treated in clinics/laboratories	Copenhageni and Brastilava
Sevá et al., 2020	São Miguel Arcanjo – SP	331	11	2015	domiciled	Cynopteri, Butembo, Grippotyphosa, Guaicura and Castellonis
	São Miguel Arcanjo – SP	373	7	2016		Cynopteri, Butembo, Hardjo (Hardjoprajitno), Grippotyphosa, Guaicura, Pomona, Copenhageni and Pyrogenes
	São Miguel Arcanjo – SP	347	14	2017		Cynopteri, Canicola, Butembo, Hardjo (Hardjoprajitno), Grippotyphosa, Guaicura, Pomona, Hebdomadis and Copenhageni
Silva et al. 2014	Alegrete - RS	5	20	uninformed	domiciled	Icterohaemorrhagiae and Canicola
	Chuí - RS	1	100			
	Cruz Alta - RS	2	0			
	Santana da Boa Vista – RS	4	0			

Silva et al., 2006	Botucatu - SP	1000	17,9	2001	domiciled	Castellonis, Autumnalis, Pyrogenes, Icterohaemorrhagiae, Canicola, Australis, Shermani, Copenhageni, Grippotyphosa, Brasiliensis, Butembo, Panama ande Wolffi
Silva et al., 2009	Botucatu - SP	1000	17,9	2001	domiciled	Castellonis, Autumnalis, Pyrogenes, Icterohaemorrhagiae, Canicola, Australis, Shermani, Copenhageni, Grippotyphosa, Brasiliensis, Butembo, Panama ande Wolffi
Silva et al., 2015	Garanhuns - PE	1	100	2012	domiciled	Patoc
	Itiquira - MT	2	50			Shermani
	Poconé - MT	7	42,86			Brastilava
	Sobral - CE	3	66,67			Copenhageni
Silva et al., 2016	Ubatuba - SP	205	14,6	uninformed	domiciled	Pyrogenes, Autumnalis, Canicola, Copenhageni, Icterohaemorrhagiae and Grypottyphosa
Silva et al., 2017	Patos - PB	39	2.6	2015	domiciled	Pomona, Bataviae, Copenhageni, Australis, Bratislava, Icterohaemorrhagiae, Autumnalis, Tarassovi, Grippotyphosa, Djasimane and Canicola
	SantaTerezinha - PB	33	15,2			
	São José de Espinhas - PB	46	13			
	São Jose do Bonfim – PB	82	12,2			
	São Mamede - PB	106	4,7			
Silva et al., 2017	Teresina - PI	425	17,41	2010-2012	shelter	Icterohaemorrhagiae, Canicola and Bratislava
Silva et al., 2018	Teresina - PI	558	13,8	2014	domiciled	Canicola, Autumnalis, Icterohaemorrhagiae, Butembo, Australis, Castellonis, Pyrogenes, Copenhageni, Grippotyphosa, Pomona and Shermani
Silvestrini et al., 2020	São Paulo - SP	19	47	2014	domiciled and treated in clinics/laboratories	Canicola, Autumnalis, Cynopteri and Copenhageni
		46	26	2015	domiciled and treated in clinics/laboratories	Pomona, Autumnalis, Cynopteri and Copenhageni
		51	29	2016	domiciled and treated in clinics/laboratories	Pomona, Autumnalis, Cynopteri and Icterohaemorrhagiae
		60	52	2017	domiciled and treated in clinics/laboratories	Australis, Autumnalis, Cynopteri and Copenhageni

		52	38	2018	domiciled and treated in clinics/laboratories	Pomona, Autumnalis, Cynopteri and Copenhageni
		22	27	2019	domiciled and treated in clinics/laboratories	Canicola, Cynopteri, Icterohaemorrhagiae and Copenhageni
Souza et al., 2016	Uberlândia - MG	27	59,25	2008-2009	domiciled	Icterohaemorrhagiae, Autumnalis, Canicola, Hardjo, Bratislava, Grippothyphosa, Pomona and Wolffi
Spanga et al., 2019	Barão de Melgaço – MT	45	17,78	2009-2012	domiciled	Australis and Icterohaemorrhagiae
	Corumbá - MT	49	12,24			Australis, Icterohaemorrhagiae and Autumnalis
	Poconé - MT	319	5,64			Australis, Grippotyphosa, Icterohaemorrhagiae, Hebdomadis, Autumnalis and Tarassovi
	Santo Antônio de Leverger - MT	16	12,5			Icterohaemorrhagiae

Table S3. Correlation between Incidence Rate and GDP, Brazil (2001 to 2020)

GDP (year) X Incidence Rate (year)	Correlation	p-valor
GDP (2002) x Incidence (2001)	0.34	<0.001
GDP (2002) x Incidence (2002)	0.33	<0.001
GDP (2003) x Incidence (2003)	0.34	<0.001
GDP (2004) x Incidence (2004)	0.34	<0.001
GDP (2005) x Incidence (2005)	0.35	<0.001
GDP (2006) x Incidence (2006)	0.34	<0.001
GDP (2007) x Incidence (2007)	0.35	<0.001
GDP (2008) x Incidence (2008)	0.35	<0.001
GDP (2009) x Incidence (2009)	0.34	<0.001
GDP (2010) x Incidence (2010)	0.36	<0.001
GDP (2011) x Incidence (2011)	0.35	<0.001
GDP (2012) x Incidence (2012)	0.37	<0.001
GDP (2013) x Incidence (2013)	0.36	<0.001
GDP (2014) x Incidence (2014)	0.35	<0.001
GDP (2015) x Incidence (2015)	0.36	<0.001
GDP (2016) x Incidence (2016)	0.34	<0.001
GDP (2017) x Incidence (2017)	0.33	<0.001
GDP (2018) x Incidence (2018)	0.34	<0.001
GDP (2019) x Incidence (2019)	0.34	<0.001
GDP (2019) x Incidence (2020)	0.28	<0.001
